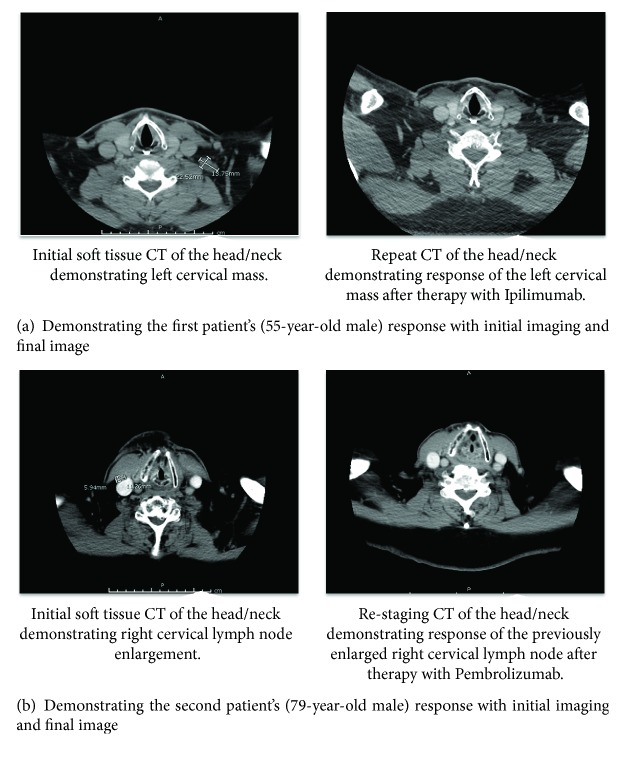# Uncommon BRAF Mutations Associated with Durable Response to Immunotherapy in Patients with Metastatic Melanoma

**DOI:** 10.1155/2017/8241624

**Published:** 2017-10-18

**Authors:** Brenen P. Swofford, Jade Homsi

**Affiliations:** ^1^University of Arizona College of Medicine-Phoenix, Phoenix, AZ, USA; ^2^University of Texas Southwestern, Dallas, TX, USA

## Abstract

Melanoma is a disease process which has been increasing in incidence over the past three decades and metastatic melanoma carries a poor prognosis. Through genetic studies of this disease, it has been determined that the BRAF V600 mutation plays a major role in the pathophysiology of the disease and this has led to the utilization of targeted therapy (BRAF and MEK inhibitors) in its treatment. Other BRAF mutations (non-V600 mutations) are rare in melanoma and targeted therapy is not indicated for patients with these mutations due to reduced response rates. An emerging option for metastatic melanoma with uncommon BRAF mutations is immunotherapy using checkpoint inhibitors such as PD-1 inhibitors or CTLA-4 inhibitors. Currently, it is unknown how patients with BRAF non-V600 mutations respond to immunotherapy. This report will examine the effect of immunotherapy on two distinct metastatic melanoma patients, each with uncommon BRAF mutations, occurring outside the V600 locus (E586K and G469E). These patients were noted to have a durable, complete response when treated with immunotherapy and continue to exhibit a response 9 and 15 months after discontinuing therapy. Further research and clinical trials are needed to study patients with uncommon BRAF mutations and the potential therapeutic benefit of immunotherapy.

## 1. Introduction

Melanoma is currently the fifth most common cancer in American men and seventh most common in American women [1]. Additionally, the incidence of this disease process is increasing dramatically [1, 2]. Patients with localized disease often only require surgical resection [2]. For those who have metastatic melanoma, it was originally a diagnosis with a poor prognosis (roughly 10% 5-year survival rate) and very limited, effective treatment options; however, with the utilization of targeted and immune therapies, the median survival time is now approaching 2-3 years [2, 3].

An expanded evaluation of molecular biology and pathogenesis of melanoma cells has led to the discovery of a new category of therapy often called targeted therapy. Available targeted therapies are directed at the mitogen-activated protein kinase (MAPK) pathway, a key signaling pathway that is activated in melanomas [4]. The serine/threonine kinases BRAF and CRAF are perhaps the most important downstream mediators of this pathway and mutations lead to clonal expansion and tumor progression [5, 6]. When activated, these kinases interact with the extracellular sign-regulated kinase (ERK), which initiates MEK phosphorylation, leading to phosphorylation of ERK and subsequent promotion of cellular growth of the tumor cells [5, 6]. BRAF mutations commonly affect exons 11 and 15 [7]. Currently, ~100 mutations of the BRAF gene have been determined to be associated with cancers, with the majority occurring at the glycine P loop (involved in stabilizing the phosphate groups of ATP during enzyme binding) and the activation segment (stabilizing the inactive form of the kinase) [8].

In patients with BRAF mutations, 80–90% contain an activating mutation occurring at the V600 locus (with the most common mutations being V600E and V600K) [3]. Vemurafenib and Dabrafenib are two targeted BRAF inhibitors that have demonstrated the ability to induce tumor regression and prolong overall survival in patients with metastatic melanoma possessing the V600 mutation [3, 6, 9]. In the BRIM-3 trial, patients with the V600 mutation experienced a significantly longer median overall survival when treated with Vemurafenib [9]. However, one associated negative finding from targeted therapies is the development of resistance to treatment by the tumor cells [9]. There are multiple factors that may be involved in the development of resistance by these tumor cells. One factor being studied is the association between resistant melanoma tumor cells and the CRAF protein [10].

Immunotherapy attempts to stimulate the immune system to destroy cells by inducing, enhancing, or suppressing the immune response to the cancer cells. In regard to melanoma, current immunotherapy options include checkpoint inhibitors (Ipilimumab, Pembrolizumab, and Nivolumab) [11]. Ipilimumab is a monoclonal antibody to CTLA-4 which augments cellular proliferation by binding to the cytotoxic T-lymphocyte associated antigen 4 (CTLA-4) [12, 13]. CTLA-4, in melanoma, is bound by tumor antigen leading to down-regulation of T-cell activation pathways [12, 13]. Ipilimumab blocks the CTLA-4 receptor allowing for enhanced T-cell activation/proliferation [12, 13]. Pembrolizumab and Nivolumab are monoclonal antibodies which inhibit programmed cell death by binding to the PD-1 receptor on T-cells [11, 14]. This binding inhibits the negative immune regulation caused by the tumor and antigen presenting cells [11, 14]. Anti-PD-1 antibodies reverse the T-cell suppression allowing an antitumor response [11, 14]. Clinical trials of immunotherapy in melanoma are still relatively new and the outcome with immunotherapy in patients with uncommon BRAF mutations is unknown. This report will examine the effect of immunotherapy on two distinct metastatic melanoma patients, each with uncommon BRAF mutations, occurring outside the V600 locus (E586K and G469E).

## 2. Cases

The first patient is a 55-year-old male with metastatic melanoma of unknown primary source diagnosed in March of 2015 after presenting with right hip pain of several weeks duration. The radiographs of the right hip revealed a 5.5 cm lytic lesion of the proximal femoral shaft and MRI further characterized the lesion as having aggressive features. Bone biopsy and pathologic evaluation demonstrated metastatic melanoma. Molecular testing revealed a BRAF E586K mutation; NRAS and C-Kit mutations were not detected. The patient underwent palliative surgery for tumor burden removal with negative margins and was started on Ipilimumab 3 mg/kg ×4 doses and palliative radiation to the affected area. The patient tolerated therapy and completed it in July 2015. He was asymptomatic and repeat imaging indicated no evidence of disease. In June of 2016 the patient presented with a left cervical mass and cervical lymphadenopathy; recurrent metastatic melanoma was confirmed by biopsy (Figure 1(a)). Given the previous response to Ipilimumab, the patient was reinitiated on this therapy ×4 cycles using the same dosage. The patient tolerated the treatment and imaging post treatment has indicated complete response to therapy (Figure 1(a)). The patient continues to demonstrate a complete response more than 9 months later.

The second patient is a 79-year-old male who was initially diagnosed with melanoma after a biopsy of the left nasal ala in April of 2014. A CT of the neck was concerning for necrotic lymph nodes on the left. Wide, local excision of the primary melanoma and left cervical lymph node dissection demonstrated a 9 mm nonulcerated melanoma and 4/25 positive lymph nodes for metastatic melanoma. He underwent adjuvant radiation therapy to the neck. Nine months later, he had a biopsy proven recurrent disease in the left and right cervical lymph nodes (Figure 1(b)). BRAF mutation testing indicated a G469E mutation and NRAS mutation was not detected. CT scans also demonstrated multiple bilateral lung nodules. The patient was initiated on Pembrolizumab 2 mg/kg every three weeks intravenously in May 2015 and staging scans in September 2015 (after 4 doses) revealed complete response to therapy in the lymph nodes and lungs (Figure 1(b)). He was continued on Pembrolizumab with the last dose occurring in May of 2016 and is being followed with close observation. The patient tolerated the treatment well and scans continue to show complete response lasting more than 15 months.

## 3. Discussion

Metastatic melanoma is a disease of increasing incidence and one of poor prognosis [2]. The BRAF V600 mutation is an important factor to direct proper treatment through targeted therapy [6]. However, for those patients with uncommon BRAF mutations (non-V600), as exhibited by the two patients presented in this case report, immunotherapy may offer a potential treatment option. Studies indicate that Ipilimumab can induce long-lasting disease control, that is not influenced by BRAF status [12, 13]. Additionally, anti-PD-1 agents such as Pembrolizumab have shown higher response rates, with most response rates lasting greater than 12 months [11, 14]. The first patient possessed a BRAF E586K mutation and was treated with Ipilimumab twice with significant disease regression and complete response radiographically noted now for 9 months. The second patient possessed a BRAF G469E mutation and was started on Pembrolizumab, with a complete response for more than 15 months, and remains off therapy. The durable response rates noted with these patients, combined with the benefits in tolerance, may represent the future of metastatic melanoma with uncommon BRAF mutation therapy.

While V600 is the most common mutation detected in patients with melanoma, more than 100 mutations on exons 11 and 15 have been reported by the Catalog of Somatic Mutations in Cancer database [7]. In regard to melanoma, data is limited but it is estimated that ~10% to as high as 30% of patients with melanoma have a non-V600 mutation [10, 15]. Current research regarding patients with non-V600 mutations is incomplete but patients with non-V600 mutations generally have a more aggressive clinical course and are not usually responsive to the selective BRAF therapy options [10, 15]. Data on the outcome of immunotherapy in patients with metastatic melanoma and uncommon BRAF mutations is unstudied.

The exact mechanism of action and tumor pathogenesis regarding immunotherapy and the durable treatment response is still being investigated. One hypothesis relates to the CRAF serine kinase. CRAF is related to the plasma membrane and can activate MAPK signaling [10]. CRAF has other functions independent of the MAPK pathway such as an association with mitochondria where it regulates apoptosis and cellular death [10]. Preliminary research of CRAF and non-V600 mutations demonstrates that, with CRAF knockdown, induced apoptosis in melanoma cells occurs [10]. It is possible that immunotherapy inhibits or down-regulates the CRAF kinase leading to apoptosis of the melanoma tumor cells. Additionally, an association between resistant melanoma tumor cells and elevated CRAF proteins is being studied [16]. It is believed that the CRAF protein decreases the bioavailability of therapeutic agents in tumor cells [16]. It is possible that immunotherapy may modulate or affect the CRAF protein levels, thereby decreasing the development of resistance as both of these patients have experienced a durable response with repeated use of their respective immunotherapy agents. Further hypotheses and clinical studies could examine uncommon BRAF mutations and tumor cells, to evaluate if these mutations yield greater production of PDL-1 ligands or decreased tumor antigen leading to a greater activity of CTLA-4 antibodies, which confers a greater response to their respective immunotherapy agents as indicated by these two cases.

Based upon the significant, durable responses experienced by the two patients presented in this case report, perhaps immunotherapy could be considered first-line therapy for patients with uncommon BRAF mutations. Further research is needed to assess the mechanism of action, evaluate for differences in outcomes with various immunotherapy options, and determine the long-term response rates/potential side effects of immunotherapy in this population.

## Figures and Tables

**Figure 1:**